# *In vivo* depletion of serum IgG by an affibody molecule binding the neonatal Fc receptor

**DOI:** 10.1038/s41598-018-23481-5

**Published:** 2018-03-23

**Authors:** Johan Seijsing, Shengze Yu, Fredrik Y Frejd, Ingmarie Höiden-Guthenberg, Torbjörn Gräslund

**Affiliations:** 1 School of Biotechnology, KTH Royal Institute of Technology, Roslagstullsbacken 21, 11417 Stockholm, Sweden; 2 Affibody AB, Gunnar Asplunds allé 24, 171 63 Solna, Sweden; 3 Present Address: Department of Molecular Biosciences, The Wenner-Gren Institute, Stockholm University, Stockholm, Sweden

## Abstract

Lowering the total level of Immunoglobulin G (IgG) in circulation is a promising general treatment option for many autoimmune diseases driven by pathogenic autoantibodies. The half-life of IgG in circulation is unusually long as a consequence of its interaction with the neonatal Fc receptor (FcRn), which protects it from lysosomal degradation by cells in contact with blood. Blocking the IgG/FcRn interaction prevents FcRn-mediated rescue, which may lead to increased catabolism and a lowering of the total IgG level. Here, we find that an engineered alternative scaffold protein, an affibody molecule, interacting specifically with FcRn, is able to block the IgG/FcRn interaction *in vitro*. The affibody molecule (Z_FcRn_) was expressed alone or as a fusion to an albumin binding domain (ABD), to extend its half-life in circulation, in both cases with retained affinity and blocking potential. Repeated i.v. injections in mice of Z_FcRn_ and Z_FcRn_-ABD were found to result in an up to 40% reduction of the IgG serum-level after 5 days. Potential applications of Z_FcRn_ as a general treatment modality for autoimmune diseases are discussed.

## Introduction

Pathogenic immunoglobulin G (IgG) autoantibodies are responsible for driving pathogenesis in a number of autoimmune diseases^1^. Compared to other serum proteins, IgG have an unusually long half-life in circulation due to interaction with the neonatal Fc receptor, which protects it from lysosomal catabolism by cells in contact with blood. In humans, the average half-life of IgG in circulation is approximately 3 weeks^2^ and in mice it is 6–8 days^3^. However, in FcRn^−/−^mice the half-life of IgG in circulation is reduced to 1 day^4^ and the mice cannot maintain IgG homeostasis, resulting in a 70–80% reduction of the total level of IgG.

FcRn is a hetero-dimeric receptor, consisting of an α-chain and β2-microglobulin (β_2_m), of which it has the latter in common with the class I Major histocompatibility complex^5^. It resides predominantly in the endosomes, where it can bind to IgG in the slightly acidic environment (pH < 6.5). FcRn together with its bound cargo is then sorted from the endosomes, followed by transport to the cell surface, where the cargo is released upon encountering the higher pH (>7) in the blood. This rescue mechanism is responsible for the long serum circulation half-life of IgG. With a similar mechanism but with a binding site that is separate from the IgG-binding site, FcRn can rescue serum albumin from lysosomal catabolism, also leading to a long residence time in circulation^6^.

Convincing evidence suggests that blocking FcRn-mediated rescue of IgG can ameliorate the symptoms of many different autoimmune diseases^7–9^. In addition, FcRn^−/−^mice have been found to be protected from induction of e. g. autoimmune arthritis, which suggest that FcRn may also play an important role in the development of different autoimmune diseases^10^. This was further supported by the finding that FcRn deficiency could protect animals in a model of the IgG-driven autoimmune disease Epidermolysis bullosa acquisita^11^. Several strategies have been evaluated for blocking the FcRn/IgG interaction towards the goal of increasing IgG catabolism in order to treat different autoimmune diseases^12^. Intravenous Ig (IVIg) is the administration of large amounts of donor-derived polyclonal IgG, and has been found to be efficient for treatment of e.g. Guillain–Barré Syndrome and is used clinically^13^. The mechanism of IVIg action is partly to increase catabolism of pathogenic IgG by blocking IgG-mediated rescue by FcRn through saturation of the rescue machinery^14^. However, IVIg treatment requires a large amount of protein making it expensive and is derived from a limited human donor source. ABDEGs (antibodies that enhance IgG degradation) are IgG molecules, where the Fc-part has been engineered to bind with high affinity to FcRn at both physiological and endosomal pH. These molecules can decrease the overall serum level of IgG in mice^15^ and have also been shown to ameliorate disease in an antibody dependent murine model of multiple sclerosis^8^. In a mouse model of arthritis, a 25 to 50 times lower amount of an ABDEG was as efficient in ameliorating the disease-symptoms compared to IVIg treatment. Monoclonal antibodies binding to FcRn have been developed and have been shown to increase IgG catabolism or have been shown efficacious in ameliorating the symptoms associated with different autoimmune diseases in animal models^9,16^. For example, a 50-fold lower amount of a β_2_m-binding monoclonal antibody was as efficient in increasing catabolism of a model antibody in mice in comparison with IVIg^16^. Overall, the antibody-based reagents blocking the IgG/FcRn interaction show promise, or in the case of IVIg is used clinically. However, a potential drawback with antibody-based reagents is their natural interaction with Ig-receptors, which could activate parts of the immune system as a side effect, which will have to be carefully monitored during clinical development of these reagents. Also, recombinant antibody-based reagents normally require an advanced mammalian production host, which makes the cost-of-goods high.

Alternatives to antibody-based reagents for blocking the IgG/FcRn interaction have been investigated. In one study, a 26 amino acid peptide (SYN1436) binding to FcRn was developed, and was shown to decrease the overall serum-level of IgG upon injection into non-human primates^17^. Its ability to ameliorate the symptoms in a passive immunization disease model of Goodpastures syndrome was also investigated^18^. In another study, small molecules interfering with the FcRn/IgG interaction were developed, however their *in vivo* function remains to be studied^19^. *In vivo* use of small peptides, such as SYN1436, or small molecules is hampered by their short residence time in circulation, requiring frequent administrations.

In this study, we have investigated a novel alternative to antibody-based reagents to block the IgG/FcRn interaction by use of an FcRn-binding affibody molecule (Z_FcRn_)^20^. Affibody molecules are affinity protein domains, 58 amino acids long, that have a folded anti-parallel three-helix bundle structure. They have been generated to bind to a variety of target proteins with high affinity and specificity^21^. We investigated if one of the previously generated affibody molecules was able to interfere with the IgG/FcRn interaction *in vitro*. After finding that the IgG/FcRn interaction could be blocked we investigated the ability of Z_FcRn_ to decrease the over-all level of IgG in circulation in mice. Similar to peptides, such as SYN1436, and small molecules, affibody molecules have a relatively short circulation residence time *in vivo*. We therefore constructed a fusion protein consisting of Z_FcRn_ and an albumin binding domain (ABD) for extension of the half-life of the fusion protein in circulation. The ABD is an engineered, independently folding domain, that can interact with high affinity with serum albumin (SA) in blood^22^, and has in several previous publications been shown to increase the circulation half-life of attached affibody molecules significantly^23,24^. Interaction with SA also endows the fusion protein with the ability to indirectly interact with the FcRn-based machinery, resulting in protection from lysosomal degradation. However the ABD does not affect SA interaction with its site on FcRn^24^, which in turn is distinct from the site of IgG and Z_FcRn_ interaction.

## Results

### Construct design

**Figure 1:**
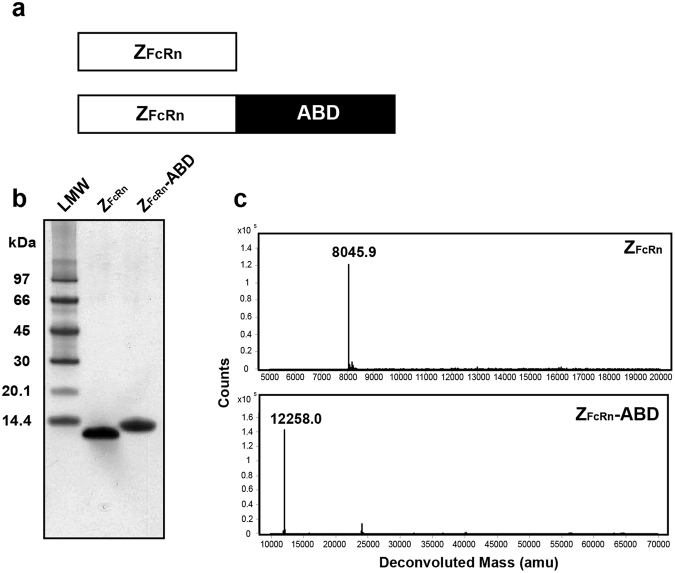
Production and initial characterization of the Z_FcRn_ constructs. Panel (**a**) schematically shows the two proteins investigated in the study. The purified proteins were analyzed by SDS-PAGE separation (**b**), where lane LMW is the separation of a molecular weight marker standard. Numbers to the left corresponds to the molecular weight of the marker proteins in kDa. Spectra from mass spectrometry analysis of the purified proteins are shown in Panel (c).

Phage display based selections were previously performed to identify several affibody variants interacting specifically with FcRn^20^. In this study, one of them (Z_FcRn_2_, herein called Z_FcRn_) was investigated for its ability to lower the total serum concentration of IgG by blocking the FcRn/IgG interaction. Since Z_FcRn_ is small (Mw 6–7 kDa) it will likely be lost during glomerular filtration in the kidneys, resulting in a short half-life in circulation. Two variants of Z_FcRn_ were therefore evaluated; one consisted of Z_FcRn_ alone and one included an albumin binding domain (ABD)^22^ for serum half-life extension (Fig. 1a). The use of the ABD has previously been found to increase the serum half-life of attached affibody molecules from 0.5 h up to 41 h^23^.

### Affibody production and initial characterization

The constructs were expressed in *Escherichia coli* and purified to homogeneity. The proteins were analyzed by SDS-PAGE (Fig. 1b, Supplementary Figure 1) followed by LC/MS analysis (Fig. 1c), which showed proteins of >98% purity with correct molecular masses. The level of potential contaminating endotoxins was measured and was found to be below the limit of detection. The tendency to precipitate was also investigated, where the proteins were frozen at −80 °C. Upon thawing no precipitation could be detected.

### Blocking the IgG/FcRn interaction *in vitro*

**Figure 2:**
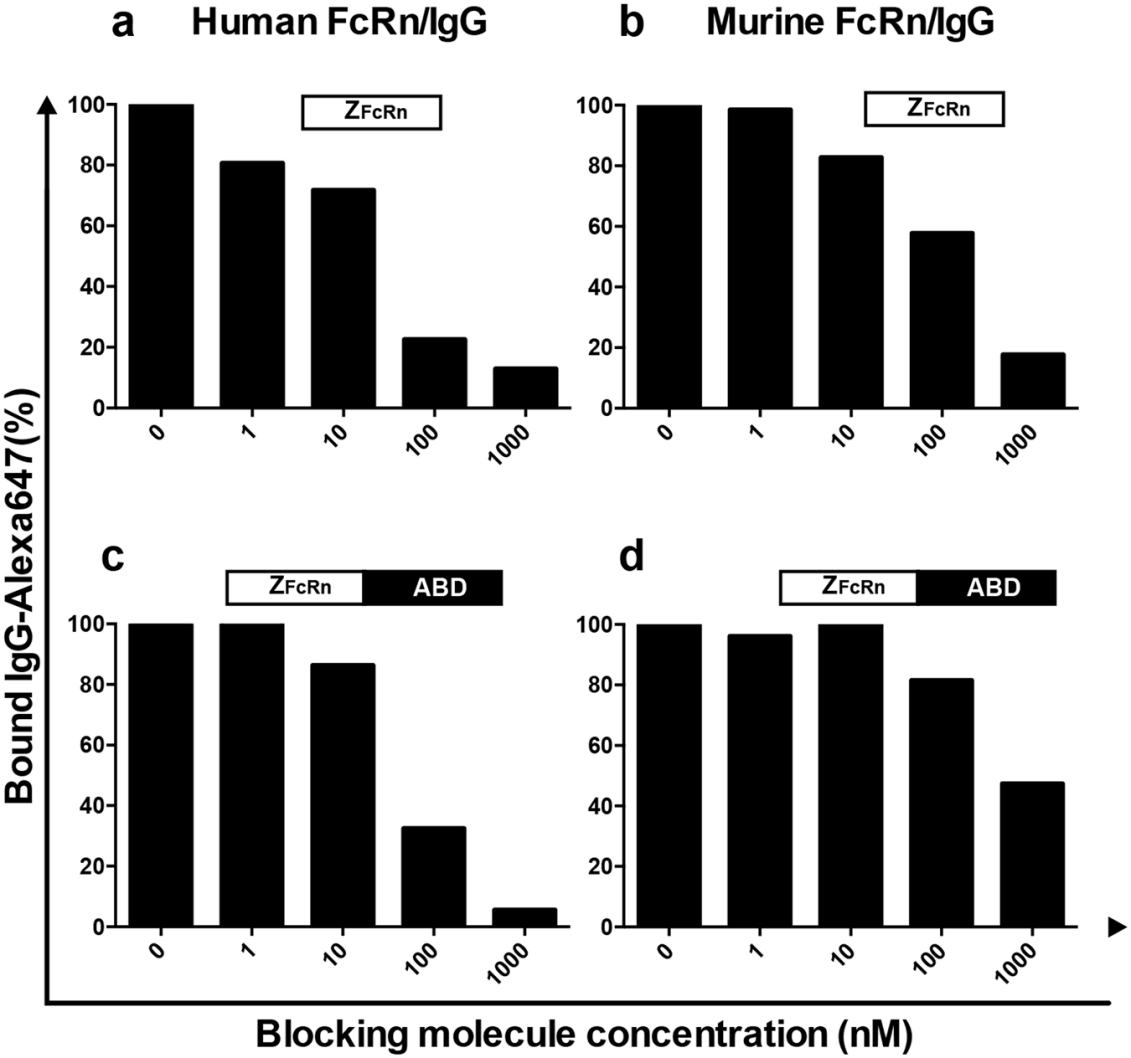
*In vitro* blocking of the IgG/FcRn interaction. HeLa cells expressing the mouse or human ortholog of FcRn as a fusion to eGFP, hFcRn-eGFP-HeLa hB2m and mFcRn-eGFP-HeLa mB2m respectively, were stained with Alexa647-labeled human or mouse IgG. During staining Z_FcRn_ or Z_FcRn_-ABD were added at different concentrations. After staining, the cells were analyzed by flow cytometry where mean fluorescence intensity (MFI) values corresponding to Alexa647-IgG fluorescence were determined. The Y-axis corresponds to the measured values as percentage of the MFI measured without addition of affibody. The X-axis corresponds to the added concentration of Z_FcRn_ or Z_FcRn_-ABD. (**a**) Cells expressing human FcRn-eGFP were stained with human IgG in the presence of Z_FcRn_; (**b**) Cells expressing mouse FcRn-eGFP were stained with mouse IgG in the presence of Z_FcRn_; (**c**) Cells expressing human FcRn-eGFP were stained with human IgG in the presence of Z_FcRn_-ABD; (**d**) Cells expressing mouse FcRn-eGFP were stained with mouse IgG in the presence of Z_FcRn_-ABD.

To investigate if Z_FcRn_ and Z_FcRn_-ABD were able to block the interaction between IgG and FcRn, a blocking assay was performed. HeLa cells recombinantly expressing either human or mouse FcRn as fusion to enhanced green fluorescent protein (eGFP) were incubated with fluorescently labeled human or murine IgG, respectively. During staining, Z_FcRn_ or Z_FcRn_-ABD was present at different concentrations to potentially block the IgG/FcRn interaction. The cells were subsequently analyzed by flow cytometry where IgG fluorescence was measured. The IgG fluorescence was found to decrease in a concentration dependent manner with an increasing concentration of Z_FcRn_ or Z_FcRn_-ABD (Fig. 2), showing that the IgG/FcRn interaction could indeed be blocked. This suggests that the proteins could potentially be used to decrease the overall level of IgG in both humans and mice. Both Z_FcRn_ and Z_FcRn_-ABD were more efficient in blocking the human IgG/human FcRn interaction (Figs 2a,c) than the mouse IgG/mouse FcRn interaction (Figs 2b,d), which suggest that the ability to block FcRn in humans should be at least equal or better than the blocking effect that will potentially be observed in mice. Also, Z_FcRn_ appeared to be somewhat more efficient than Z_FcRn_-ABD in blocking both interactions, except for the highest concentration of Z_FcRn_-ABD where the construct was more efficient in blocking the human IgG/human FcRn interaction than Z_FcRn_ at the same concentration.

### Detailed characterization of affinities to FcRn and serum albumin

**Figure 3:**
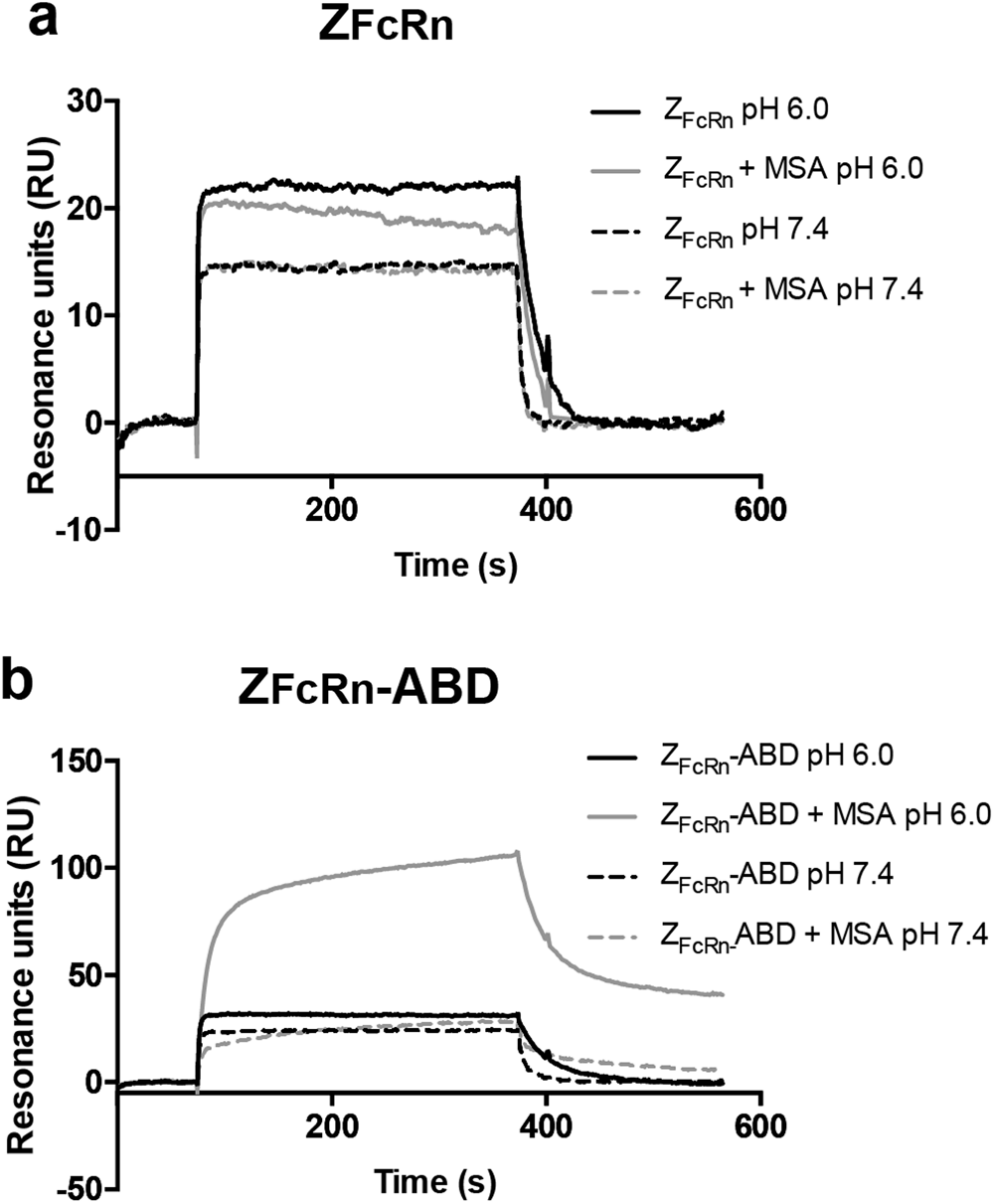
Interaction of Z_FcRn_ constructs with FcRn. The interaction of Z_FcRn_ and Z_FcRn_-ABD with human FcRn at different pH and in the presence or absence of SA was investigated by biosensor analysis. The panels show overlays of representative sensorgrams recorded after injection of Z_FcRn_ (**a**) and Z_FcRn_-ABD (**b**).

A detailed characterization of the interactions of Z_FcRn_ and Z_FcRn_-ABD with both FcRn and serum albumin were conducted by biosensor analysis. First, Z_FcRn_ and Z_FcRn_-ABD were injected over a surface with immobilized human FcRn at pH 6.0 and 7.4 in the presence or absence of mouse serum albumin (Fig. 3). The equilibrium response when injecting Z_FcRn_ was appreciably higher at pH 6.0 than at pH 7.4 suggesting a higher affinity at pH 6.0 (Fig. 3a). The equilibrium response was largely unaffected by the presence of MSA, which was expected since MSA should not interact with Z_FcRn_ and its interaction with human FcRn at the concentration used is below the limit of detection in the assay. A control experiment where only MSA at the same concentration was injected over the surface gave no detectable response (Supplementary Figure 2). The equilibrium response when injecting Z_FcRn_-ABD was similarly higher at pH 6.0 than at 7.4 also suggesting a higher affinity at 6.0 (Fig. 3b). Here the presence of MSA resulted in an increase in the equilibrium response and a decrease in the on-rate, which is indicative of a larger complex interacting with the surface, suggesting that the complex Z_FcRn_-ABD/MSA is able to interact with FcRn.

**Figure 4:**
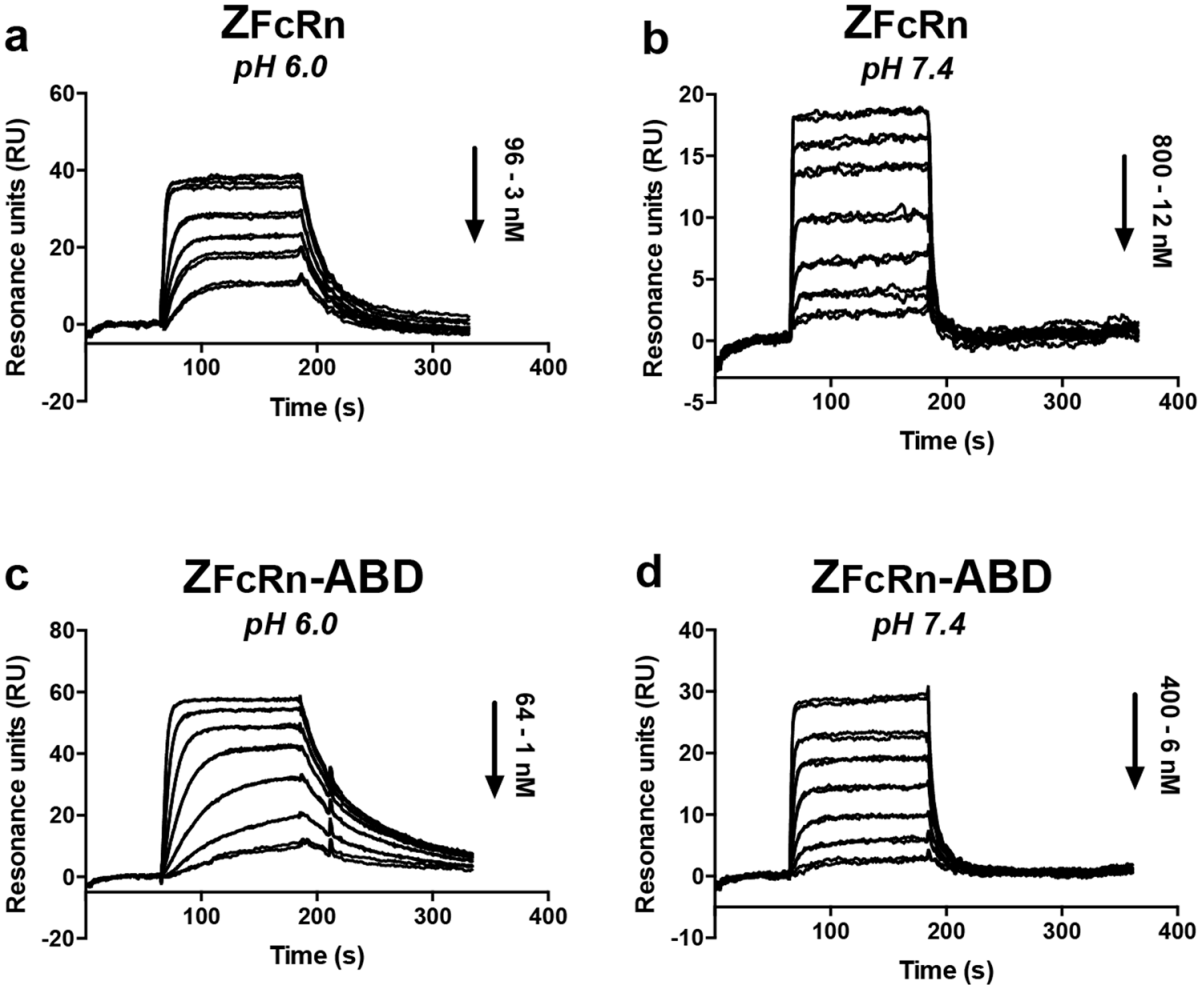
Determination of the equilibrium dissociation constant for the Affibody/FcRn interactions. The interaction between Z_FcRn_ or Z_FcRn_-ABD and human FcRn was analyzed at different pH by injection of dilution series of the proteins over a sensor chip with immobilized human FcRn. (**a**) A dilution series of Z_FcRn_ at pH 6.0 was injected over the surface; (**b**) A dilution series of Z_FcRn_ at pH 7.4 was injected over the surface; (**c**) A dilution series of Z_FcRn_-ABD at pH 6.0 was injected over the surface; (**d**) A dilution series of Z_FcRn_-ABD at pH 7.4 was injected over the surface. Each injection was done in duplicates and the panels show an overlay of the duplicate experiments.

The affinities to FcRn were also determined by injecting dilution series of Z_FcRn_ and Z_FcRn_-ABD at pH 6.0 and 7.4 (Fig. 4, Table 1). The affinity of Z_FcRn_ was found to be approx. 40 times stronger at pH 6.0 compared to pH 7.4 (K_D_: 9 nM versus 400 nM; Figs 4a,b). Similarly, the affinity of Z_FcRn_-ABD was approximately 10 times stronger at pH 6.0 compared to pH 7.4 (K_D_: 3 nM versus 40 nM; Figs 4c,d). The difference in affinity between Z_FcRn_ and Z_FcRn_-ABD at pH 6.0 is within the margin of error, with a tendency for a higher affinity for the ABD-tagged construct. At pH 7.4 the difference in affinity between Z_FcRn_ and Z_FcRn_-ABD is ten-fold. In conclusion, the experiment shows that expression of Z_FcRn_ as a fusion to the ABD does not affect its interaction with FcRn. On the contrary, the affinity appears to be slightly stronger.

**Table 1:** Affinities to FcRn and MSA.

Construct	Target	K_D_ (nM)	K_D_ (nM)
		pH 6.0	pH 7.4
Z_FcRn_	hFcRn	8.7	365
	MSA	N.B.^a^	N.B.
Z_FcRn_-ABD	hFcRn	3.3	36
	MSA	0.3	0.5

^a^N.B. = No detectable binding.

**Figure 5:**
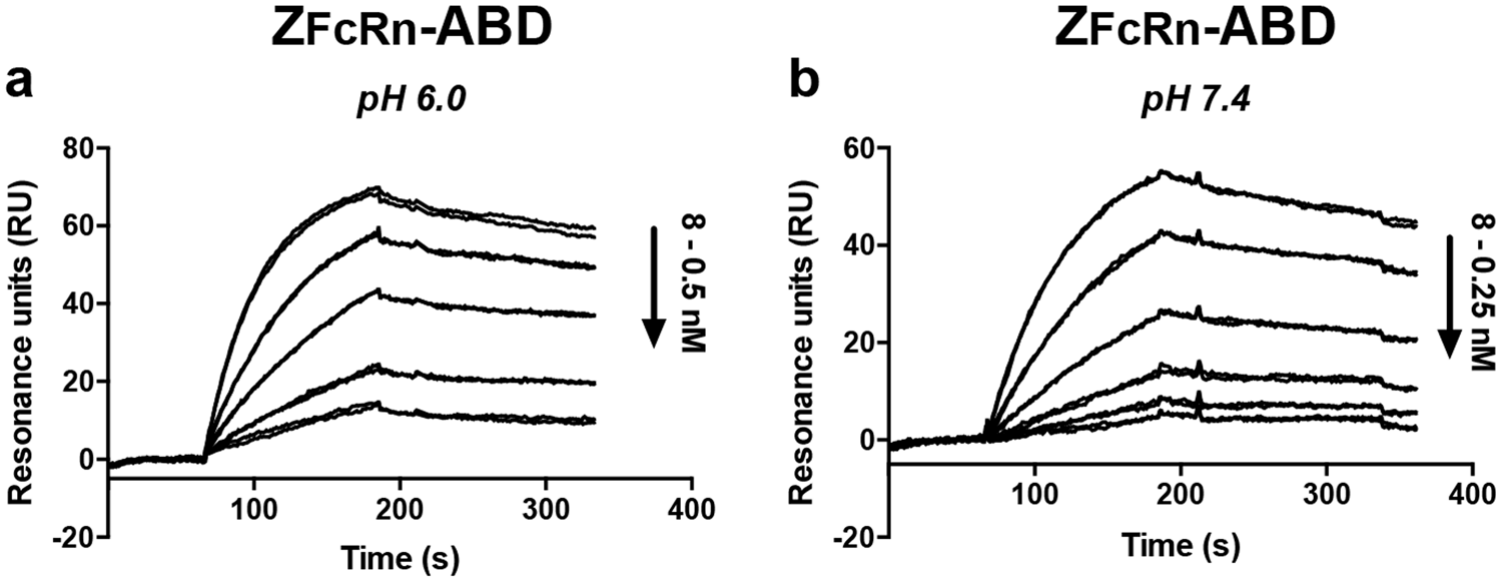
Determination of the equilibrium dissociation constant for the Affibody/SA interactions. The interaction between Z_FcRn_-ABD and SA was analyzed at different pH by injection of dilution series of the protein over a sensor chip with immobilized human SA. (**a**) A dilution series was injected over the surface at pH 6.0; (**b**) A dilution series was injected over the surface at pH 7.4. Each injection was done in duplicates and the panels show an overlay of the duplicate experiments.

The affinity to MSA was determined by injecting dilution series of Z_FcRn_-ABD at pH 6.0 and 7.4 (Fig. 5, Table 1). The affinities were found to be similar at both pH-values, 0.3 nM and 0.5 nM, respectively.

### Reduction of endogenous murine IgG *in vivo*

**Figure 6:**
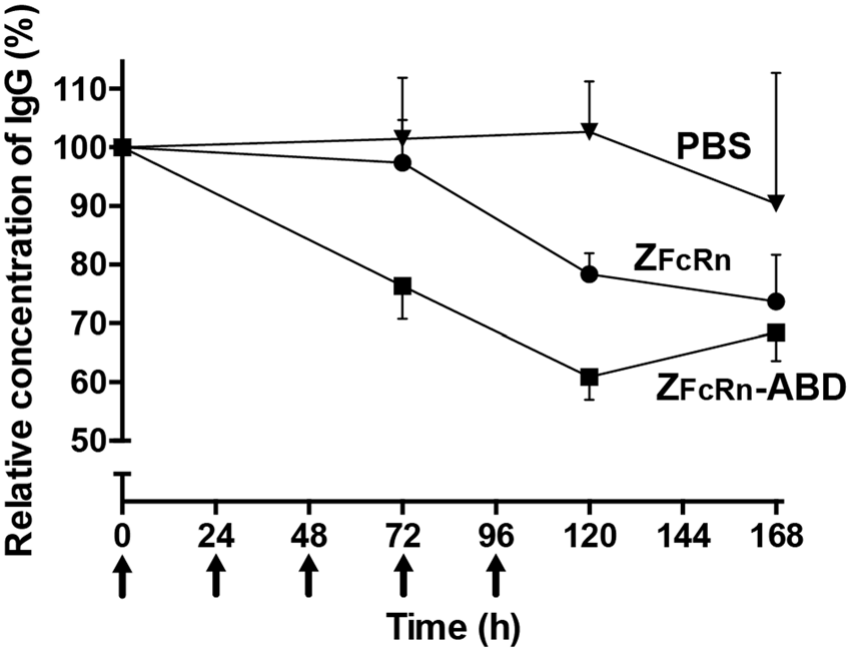
*In vivo* reduction of total IgG. Determination of the serum concentrations of IgG after repeated injections (arrows) of Z_FcRn_, Z_FcRn_-ABD or PBS (vehicle). Each data-point represents the average of values obtained from seven animals and the error-bars correspond to SD.

The finding that Z_FcRn_ and Z_FcRn_-ABD could block the human as well as the mouse IgG/FcRn interaction merited evaluation of their potential to lower the serum concentration of IgG in mice. Z_FcRn_, Z_FcRn_-ABD or PBS-buffer (vehicle) was administered to mice once daily during five days by i.v. injection and the serum concentration of IgG was determined by ELISA as a function of time (Fig. 6). A significant reduction of the serum concentration of IgG was observed after injection of either construct, where Z_FcRn_-ABD was more efficiently reducing the IgG concentration than Z_FcRn_. For both constructs, the maximum reduction was achieved after 120 h where Z_FcRn_ reduced the serum concentration of IgG with 21.7 ± 8.9% and Z_FcRn_-ABD with 39.0 ± 10.3%. No signs of adverse effects in the animals such as weight loss were noticed during the experiment.

## Discussion

In this study, a small non-immunoglobulin based alternative scaffold protein (Z_FcRn_) was investigated for its ability to interfere with the IgG/FcRn interaction and reduce the total serum concentration of IgG in mice. This affinity protein domain, so called affibody molecule, was previously generated by selection from a library displayed on phage^20^. During affibody generation, the selection was carried out to enrich for variants binding to epitopes on the α-chain that were separate from the SA binding interface. Among several variants with specificity for FcRn, at least one was found that interferes with the IgG/FcRn interaction.

Z_FcRn_ was expressed as a single domain or as a fusion to an albumin binding domain (ABD), which was added to extend the half-life in circulation of Z_FcRn_. The affinities for FcRn were similar at pH = 6.0 but were tenfold stronger for Z_FcRn_-ABD at pH = 7.4. It is possible that the difference in affinity at pH = 7.4 contributes to the higher efficiency of Z_FcRn_-ABD to lower the serum IgG concentration *in vivo*, since it masks the receptor for IgG-interaction to a greater extent. However, rescue from degradation by FcRn occurs in the endosomes at pH = 6.0, where the affinities of the affibody variants are similar, so it is likely that the contribution to IgG-depletion in serum from the stronger affinity at pH = 7.4 is minor. A more likely explanation for the difference in efficiency between the constructs is the difference in half-life in circulation, where affibody molecules by themselves have a half-life of less than 1 h in comparison with ABD-tagged constructs that have a half-life of 35–40 h in mice^23^. Since affibody administration occurred only every 24 h, the serum concentration of Z_FcRn_ was likely low during a greater part of this time, and any FcRn-mediated rescue from degradation by cells of the vascular endothelium was thus likely minor, as previous biodistribution studies of other affibody molecules have shown that this molecular class is mainly eliminated by the kidneys and not by degradation by cells of the vascular endothelium^25^.

Decreasing the total IgG and thereby the level of pathogenic IgG in circulation is a treatment modality for many autoimmune diseases. There are different strategies to achieve a decrease in total IgG including plasma exchange, IVIg and targeting CD20(+) B-lymphocytes with a monoclonal antibody such as rituximab. The latter is for example used to treat rheumatoid arthritis patients that have failed TNF-blocking therapy^26^. B-cell depletion treatment is generally considered safe although a tendency of a higher incidence of infections compared to control has been reported in some studies^27^. IVIg is used for treatment of several autoimmune diseases, here copious amounts of IgG (1–2 g per kg body weight per injection) is administered to the patients. This option is for example used for some Guillain-Barré syndrome patients^28^. The mechanism of this treatment is still under debate^29^ and the cost is rather high as a consequence of the large amount of IgG injected. Compared to monoclonal antibodies that target and mediate killing of CD20(+) B-cells, blocking FcRn-mediated rescue of IgG is safer and can be ceased, followed by a quick repopulation of endogenous IgG production in the patient, if side effects are observed. However, IVIg has its limitations and several potential alternatives have recently been presented.

An antibody generated to interact with the α-chain of rat FcRn at pH 6.0 and 7.4 (1G3), were found to reduce the level of endogenous IgG in rats by 40% upon two consecutive injections of 30 mg/kg^9^. In the present study, a similar reduction was achieved after injection of a larger amount of Z_FcRn_. In another study^15^, the Fc region of IgG1, engineered for increased affinity to FcRn both at pH 6.0 and 7.2, was shown to give a reduction of the serum IgG-level where higher affinity resulted in higher efficacy. Both studies point towards that a combination of high affinity at pH 6 and pH 7.4 results in more efficient reduction of the serum level of IgG. However, a potential risk with using an Fc-based strategy is that it may trigger unwanted side effects of the immune system due to effector functions triggered by Fc. A non-Fc-based FcRn-blocker, such as the one presented in this study, is safer in this respect. An FcRn blocking peptide selected from a combinatorial library displayed on phage, have also been investigated previously^17^. Similar to Z_FcRn_, i.v. administration of this peptide to mice resulted in reduction of the serum IgG-level. In the same article, the authors also showed that the peptide could lower the total IgG-level in cynomolgus monkeys; further strengthening the hypothesis that FcRn-mediated blocking is a viable strategy for reduction of the serum IgG-level in humans. However, a small peptide has the drawback of a short half-life in circulation, which needs to be extended by for example the ABD-technology used in this study, to be practically usable.

Potential side effects of lowering the total level of IgG by Z_FcRn_ or the molecules described above can at present only be speculated on, but it is possible that the side effects will be similar to those found in patients with hypogammaglobulinaemia, where the IgG level is lower than normal. In a review of patients treated with rituximab, a monoclonal antibody targeting and mediating killing of CD20( + ) B-cells, several patients with hypogammaglobulinaemia could be identified^30^. Common among them was that they were more succeptible to viral, bacterial and fungal infections, mostly in the respiratory tract but also in the ear and urinary tract.

Efficient blocking of FcRn in an animal or patient requires injection of a large amount of blocking molecule, as shown in the studies above on 1G3 and ABDEGs. Even though an even larger amount was injected in the present study, it might be possible to develop FcRn-interacting affibody molecules with stronger affinity that requires injection of a smaller amount. In addition, the molecular weight of Z_FcRn_-ABD is only 13 kDa, while the molecular weight of Fc is 50 kDa and a full IgG is 150 kDa^9^, which means that the injection volume per mole is much smaller for Z_FcRn_. It should also be noted that Z_FcRn_ shows higher affinity towards the human FcRn than the murine counterpart, which implies that it may have a better effect in humans than in mice.

In conclusion, in this study we demonstrate for the first time that an alternative scaffold protein, the affibody molecule Z_FcRn_, has the ability to block the IgG/FcRn interaction *in vitro* and *in vivo*. Administration of Z_FcRn_ as a fusion with ABD to mice resulted in a significant reduction of the serum concentration of endogenous IgG. Proteins such as Z_FcRn_-ABD might be useful as a general treatment modality for IgG-driven autoimmune diseases and the results presented merits further investigation of Z_FcRn_ in different animal models of autoimmune diseases.

## Materials and Methods

### General

All chemicals and reagents were purchased from Sigma-Aldrich (Stockholm, Sweden) unless otherwise stated.

### Cloning, expression, purification and characterization of affibody constructs

The genes encoding Z_FcRn_ and Z_FcRn_-ABD were derived from Z_FcRn_2_^20^. They were assembled by PCR amplification, where a N-terminal hexahistidine-tag was added and the constructs were sub-cloned into a pET-vector backbone under control of the T7-promoter (Novagen, Darmstadt, Germany). The integrity of the constructs was verified by DNA sequencing. After transformation to *Escherichia coli* BL21(DE3) (Novagen), both constructs were expressed at 37 °C upon addition of 1 mM Isopropyl β-D-1-thiogalactopyranoside. The cultures were harvested by centrifugation. The cell pellet from Z_FcRn_-ABD production was resuspended in 1xTST (50 mM Tris; 0.2 M NaCl; 0.05% Tween-20; pH 8.0) supplemented with Benzonase (15 U/ml) (Merck, Darmstadt, Germany) and the cells were lysed by sonication. Z_FcRn_-ABD was thereafter recovered by anti-ABD agarose chromatography according to the manufacturer’s recommendations (Affibody, Solna, Sweden). The pellet from Z_FcRn_ production was resuspended in 10 mM Tris (pH 8.0) supplemented with Benzonase (15 U/ml). The solution was heated at 87 °C for 15 min to lyse the cells and denaturate endogenous *E. coli* proteins. The lysate was cleared by centrifugation and fractionated by anion exchange chromatography (Q Sepharose FF resin) with 10 mM Tris (pH 8.0) as running buffer and elution by a gradient from 0 to 1 M NaCl. Both proteins were further purified by reverse phase chromatography, employing a Source 30RPC matrix (GE Healthcare, Danderyd, Sweden). The running buffer was 10% acetonitrile in water supplemented with 0.1% trifluoroacetic acid. Elution was performed by an acetonitrile gradient from 10 to 50%. Both proteins were further purified by gel filtration using a Sephadex G25 column (GE Healthcare) with phosphate buffered saline as running buffer. The proteins were concentrated in Amicon Ultra 3 kDa MWCO filters (Millipore) and endotoxins were removed by EndoTrap red (Hyglos, Bernried am Starnberger See, Germany). Purified proteins were analyzed by LC/MS on an 1100 LC-MSD (Agilent, CA, USA). Endotoxin content was determined for both proteins using EndoLISA (Hyglos) and was found to be under the limit of detection.

### Cell culture

HeLa cell lines, expressing full-length versions of the mouse or human FcRn as fusion to eGFP has previously been created, denoted hFcRn-eGFP-HeLa-hB2m and mFcRn-eGFP-HeLa-mB2m, respectively^31^. All cell culture media and reagents were from Invitrogen or Sigma Aldrich. Cells were cultivated in a 5% CO_2_ atmosphere in a humidified incubator at 37 °C. The HeLa cell lines were grown in Eagle’s Minimum Essential Medium supplemented with 10% Fetal Bovine Serum and an Antibiotic/Antimycototic Solution.

### Blocking of IgG binding to FcRn expressed on HeLa cells

hFcRn-eGFP-HeLa-hB2m and mFcRn-eGFP-HeLa-mB2m were harvested by trypsination and washed twice with PBS (137 mM NaCl; 3 mM KCl; 8 mM Na_2_PO_4_; 2 mM KH_2_PO_4_) at pH 6.0. 100,000 cells were distributed per well in a V-bottom 96 well plate (Nunc, Rockford, IL, USA) and the cells were pelleted by centrifugation. The cells were fixed with 2% formaldehyde in PBS at pH 6.0 for 10 min at r.t., followed by washing with PBS (pH 6.0), saturated with casein (PBSC). The cells were labeled with a mix of 100 nM Alexa647-conjugated human or mouse IgG (Jackson laboratories, Bar Harbor, ME, USA) and 1000, 100, 10, 1 and 0 nM Z_FcRn_ or Z_FcRn_-ABD diluted in PBSC (pH 6.0) supplemented with 0.1% saponin (AppliChem, Darmstadt, Germany) for 1 h at 37 °C with shaking. Cells were washed with PBSC (pH 6.0) and resuspended in PBS (pH 6.0) supplemented with 1% BSA. Cells were analyzed using a Gallios Flow Cytometer (Beckman Coulter, Bromma, Sweden) and mean fluorescence intensity originating from IgG-Alexa647 was recorded. The background MFI of HeLa cells not expressing FcRn but labeled with IgG-Alexa647 was subtracted from all values. The resulting MFI-values were divided with the value obtained for IgG-Alexa647 without addition of affibody.

### SPR analysis

The affinities between murine serum albumin (MSA), human FcRn_ECD_ (hFcRn) and Z_FcRn_, expressed as monomer or as fusion to ABD, were measured by biosensor analysis on a Biacore 3000 instrument (GE Healthcare). MSA and hFcRn was immobilized on separated flow cells on a CM5 chip in acetate buffer at pH 4.65. The immobilization level was ∼600 RU for MSA and ~450 RU for hFcRn. A reference flow cell was created by activation and deactivation. McIlvaine’s phosphate-citrate buffer (pH 6.0 or 7.4), supplemented with 0.05% Tween-20, was used as running buffer and for dilution of the analytes. All analyses were performed at 25 °C with a flow rate of 50 μL/min.

The affinity constant of the different interactions was determined by injecting dilution series of the analytes as shown in the figures. The affinities were derived using BIAevaluation software (GE Healthcare), using a 1:1 Langmuir binding model. For co-binding analysis at different pH as shown in Fig. 3, 200 nM Z_FcRn_ or Z_FcRn_-ABD was injected, with or without previous incubation (4 h) with 400 nM MSA, over the flow cell with immobilized hFcRn.

### Reduction of endogenous murine IgG *in vivo*

The study, including the experimental protocol, was granted permission (N81/14) by the Regional Animal Experimental Ethics Committee in Stockholm (North) and performed by Adlego Biomedica (Uppsala, Sweden). The experiments were planned and performed in accordance with national legislation on laboratory animals’ protection. Male NMRI mice (Charles River, Köln, Germany) were divided into groups of seven animals. Z_FcRn_ or Z_FcRn_-ABD were administered in the tail vein (48 µmol/kg body weight) at five time points; 0, 24, 48, 72 and 96 h. Blood samples (80 µl) were drawn from the saphenous vein from each animal at the time points 0, 72, 120 h and by heart puncture (400 µl) at 168 h. At 0 and 72 h the sampling was performed before protein administration. Blood was left at r.t. for at least 30 min and then subjected to centrifugation before collection of serum. Isofluorane anesthesia was used at each administration and sampling, and after the last blood sampling the mice was euthanized by cervical dislocation.

The concentration of IgG in the serum samples was analyzed by a Mouse IgG ELISA kit (Mabtech, Stockholm, Sweden), essentially according to the manufacturer’s instruction. Half-area 96-well ELISA plates (Corning, NY, USA) were coated with anti-IgG antibody followed by washing with PBS and blocking with BSA in PBS. After blocking, IgG standard samples and serum samples (diluted 24300 times) were added to the plate. The plate was incubated for 1.5 h and after washing with PBS, bound IgG was detected with anti-IgG-conjugated alkaline phosphatase. The wells were developed using p-nitrophenyl-phosphate substrate for 40 min after which the absorbance at 405 nm was recorded. The concentration of IgG at each time point was calculated by dividing the measured IgG concentration with the IgG concentration at time = 0 h for the same animal, and expressing the result as “relative concentration of IgG (%)”.

## Electronic supplementary material

Supplementary information
