# Supplementary material for: In vivo depletion of serum IgG by an affibody molecule binding the neonatal Fc receptor
Source: Sci Rep. 2018 Mar 23;8:5141. doi: 10.1038/s41598-018-23481-5 (PMC5865129; doi:10.1038/s41598-018-23481-5)

In vivo depletion of serum IgG by an affibody molecule binding the neonatal Fc receptor.

Johan Seijsing, Shengze Yu, Fredrik Y Frejd, Ingmarie Höiden-Guthenberg, Torbjörn Gräslund

Legend to supplementary figures

Supplementary figure 1. SDS-PAGE separation of Z_{FcRn} (lane 1) and Z_{FcRn}-ABD (lane 2) after purification. Lane M corresponds to the molecular weight marker.

Supplementary figure 2. Interaction of MSA with FcRn. The interaction of MSA with human FcRn at different pH was investigated by biosensor analysis. The panels show overlays of representative sensorgrams recorded after injection of 400 nM at pH 6.0 and 7.4.

Supplementary figure 1

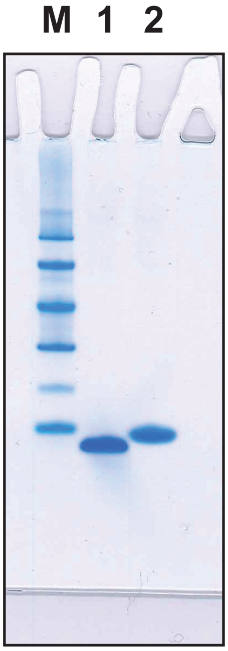

Supplementary figure 2

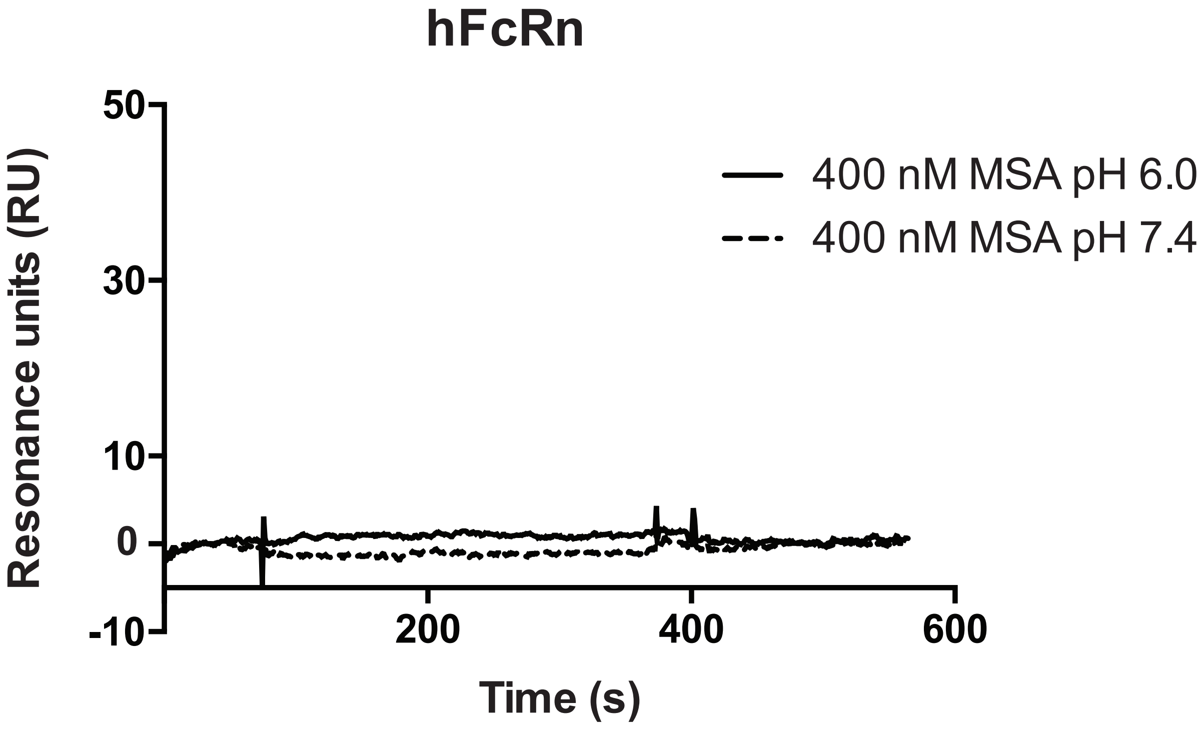

Supplement: Supplementary file 1 — Supplementary information [file 41598_2018_23481_MOESM1_ESM.pdf]
